# *Alu* insertion variants alter mRNA splicing

**DOI:** 10.1093/nar/gky1086

**Published:** 2018-11-10

**Authors:** Lindsay M Payer, Jared P Steranka, Daniel Ardeljan, JaNiece Walker, Kathryn C Fitzgerald, Peter A Calabresi, Thomas A Cooper, Kathleen H Burns

**Affiliations:** 1 Department of Pathology, Johns Hopkins University School of Medicine, Baltimore, MD 21205, USA; 2 McKusick-Nathans Institute of Genetic Medicine, Johns Hopkins University School of Medicine, Baltimore, MD 21205, USA; 3 Department of Biology, Xavier University of Louisiana, New Orleans, LA 70125, USA; 4 Department of Neurology, Johns Hopkins University School of Medicine, Baltimore, MD 21205, USA; 5 Solomon Snyder Department of Neuroscience, Johns Hopkins University School of Medicine, Baltimore, MD 21205, USA; 6 Department of Pathology and Immunology, Baylor College of Medicine, Houston, TX 77030, USA; 7 Department of Molecular and Cellular Biology, Baylor College of Medicine, Houston, TX 77030, USA; 8 Department of Molecular Physiology and Biophysics, Baylor College of Medicine, Houston, TX 77030, USA; 9 Sidney Kimmel Comprehensive Cancer Center, Johns Hopkins University School of Medicine, Baltimore, MD 21205, USA

## Abstract

RNA splicing is a highly regulated process dependent on sequences near splice sites. Insertions of *Alu* retrotransposons can disrupt splice sites or bind splicing regulators. We hypothesized that some common inherited polymorphic *Alu* insertions are responsible for splicing QTLs (sQTL). We focused on intronic *Alu* variants mapping within 100 bp of an alternatively used exon and screened for those that alter splicing. We identify five loci, 21.7% of those assayed, where the polymorphic *Alu* alters splicing. While in most cases the *Alu* promotes exon skipping, at one locus the *Alu* increases exon inclusion. Of particular interest is an *Alu* polymorphism in the *CD58* gene. Reduced *CD58* expression is associated with risk for developing multiple sclerosis. We show that the *Alu* insertion promotes skipping of *CD58* exon 3 and results in a frameshifted transcript, indicating that the *Alu* may be the causative variant for increased MS risk at this locus. Using RT-PCR analysis at the endogenous locus, we confirm that the *Alu* variant is a sQTL for *CD58*. In summary, altered splicing efficiency is a common functional consequence of *Alu* polymorphisms including at least one instance where the variant is implicated in disease risk. This work broadens our understanding of splicing regulatory sequences around exons.

## INTRODUCTION

Alternative splicing of transcripts is pervasive in the genome, with >95% of genes generating more than one mRNA by alternative splicing (1). This provides flexible substrates for gene and protein evolution (e.g. (2–4)) and enhances the cellular and functional complexity in eukaryotes (reviewed in (5,6)). In addition to enhancing mRNA and protein diversity, alternative splicing can also regulate the levels of protein produced by incorporating or excluding a stop codon in the processed transcript or shifting the protein open reading frame. When splicing is skewed beyond normal alternative splicing, the aberrant resultant isoform can result in a disease phenotype. In between these two extremes, it is possible that common variants subtly alter mRNA splicing with modest phenotypic outcomes, such as increasing common disease risk (7). Here, we investigated whether commonly occurring polymorphic *Alu* elements can alter splicing and affect mRNA isoform prevalence. In particular, these structural variants exist in the population with both the *Alu*-containing allele and the pre-insertion (no *Alu*) allele present (e.g. (8)).

*Alu* elements are short interspersed elements (SINE) that have proliferated in primate genomes through a ‘copy-paste’ mechanism of retrotransposition. There are over 1.1 million *Alu* elements in the human genome (9). *Alu* elements are ∼300 bp in length (approximately 280 bp plus a poly A tail) and are derived from 7SL RNA (reviewed in (10)). *Alu* elements can profoundly affect mRNA splicing. *De novo Alu* insertions at splice acceptor and splice donor sites can disrupt splicing and result in disease-causing alleles (11–14). A subset of *Alu* elements that have become fixed in human populations (i.e. homozygous present insertions in all individuals) can contribute to natural mRNA isoform diversity. Exonization of all or part of an *Alu* sequence is well documented at many loci and frequently occurs because *Alu* elements anti-sense to a gene can provide splice acceptor sites (e.g. (15,16)). *Alu* elements can also affect mRNA splicing isoforms by creating circular RNAs (17,18) or in ectopic assays by altering recognition of splice sites (19,20).

As new *Alu* insertions caused by retrotransposition occur at essentially random locations with respect to exons, it is notable that there is a depletion of *Alu* elements within 100 or 150 bp of an exon (20,21). This suggested to us that purifying selection eliminates those *Alu* insertions positioned to affect mRNA splicing. We hypothesized that polymorphic *Alu* insertions close to exons would be likely to affect mRNA splicing, potentially with phenotypic consequences. In this study, we identify reported polymorphic *Alu* elements mapping to this underrepresented zone near exons and test their impact on mRNA splicing. Using a splicing reporter assay to evaluate 23 loci, we find five where the *Alu* affects mRNA isoform representation by promoting either exon skipping or inclusion.

## MATERIALS AND METHODS

### Intronic *Alu* distribution

Polymorphic *Alu* elements with insertion sites mapped to single base pair resolution (8,22) were narrowed to those that map within annotated RefSeq genes (GRCh37/hg19, UCSC genome browser). All *Alu*Y elements were obtained from UCSC genome browser RepeatMasker (23) track (GRCh37/hg19, UCSC genome browser). Genome-wide exon coordinates and type were obtained from HEXevent (24). Exons were defined as alternatively spliced if the exon is skipped in any reported transcript isoform, or alternative 5′ and/or 3′ ends were reported for the exon (24). The distance between each *Alu* element and the nearest exon was determined using bedtools closest tool (25). Distribution of either *Alu*Y or polymorphic *Alu* elements was graphed as the total number of elements mapping within 25 bp bins over the total number of intronic elements.

### Ectopic minigene reporter assays

The pSpliceExpress vector ((26), Addgene) contains two constitutive rat insulin exons flanking a an intron modified for Gateway cloning (Invitrogen). For each tested locus, the alternatively used exon and ∼2000 bp of flanking intronic sequence was amplified using primers listed in Supplementary Table S1. The region was amplified from DNA of Centre d'Étude du Polymorphisme Humain (CEPH) Utah Residents with Northern and Western European Ancestry (CEU) (Coriell Institute for Medical Research, Camden, NJ, USA) using individuals who were either homozygous for the *Alu* insertion or for the empty (non-*Alu* containing) allele. These fragments were cloned into the minigene reporter intron using Gateway cloning (Invitrogen) and Sanger sequence verified to ensure no other sequence difference existed outside of the *Alu* genotype. Two independent clones with the polymorphic *Alu* present and two constructs without the *Alu* present were generated and sequence verified. Each vector was transfected into 293T cells using Fugene HD (Promega) with manufacturer's suggested protocol. After 24 h, RNA was isolated using Quick RNA MicroPrep Kit (Zymo Research) per manufacturer's suggested protocols. cDNA was synthesized with iScript cDNA Synthesis Kit (BioRad) per manufacturer's protocol using 1 μg of RNA and random primers. PCR was performed with primers binding within the rat insulin exons (5′-CAGCACCTTTGTGGTTCTCA-3′, 5′-AGAGCAGATGCTGGTGCAG-3′). The relative quantification of alternatively spliced RNA isoforms was performed on ethidium bromide stained agarose gels with band intensities normalized for DNA fragment length. A representative gel image with the independent clones is shown for each locus. Two separate transfections were performed for each independent clone. For each locus, we performed ANOVA analysis and the only variable with significant differences was the *Alu* genotype. Therefore, quantifications from the two independent clones and their replicates were combined resulting in four data points for each type of construct (i.e. with or without *Alu*) for each locus tested. The quantification of each clone from each experiment is available in Supplemental Figure S1. Quantification is graphed as percent of transcripts that skip the alternative exon. Unpaired *t*-tests were used to compare the percent exon skipping for each construct type for a given locus and adjusted for multiple comparisons when necessary as described below.

To define further the mechanism by which the *Alu* polymorphism alters splicing, additional constructs were generated for the *CD58* and *SLC2A9* loci. These sequences included one of the following being cloned in place of the polymorphic *Alu* sequence: *Alu*Ya5 consensus sequence with an average length polyA tail (Supplementary Table S2), randomized *Alu*Ya5 consensus sequence (with the polyA tail sequence scrambled with the rest of the *Alu* sequence), or randomized sequence with GC content matching the pre-insertion integration site (the same length as the polymorphic *Alu* including the polyA tail) (Supplementary Table S2). For all randomized sequences, two different sequences were evaluated at each locus to decrease the potential to confound results by introducing cryptic regulators. When results differed for the scrambled *Alu* sequence at *CD58*, a third scrambled sequence was also evaluated (Supplementary Table S2). These sequences were synthesized (Invitrogen) and cloned in place of the polymorphic *Alu* element in the vector using Gibson Assembly (New England BioLabs). Final vectors were sequence verified to ensure seamless replacement of the polymorphic *Alu* sequence with the desired synthesized fragments. Because seven or eight constructs were evaluated at *SLC2A9* and *CD58* respectively, the *P*-value of 0.05 was adjusted to 0.0024 or 0.0019 to account for the 21 or 26 unpaired *t*-tests that were performed.

### Additional analysis at *CD58* locus

Genotyping and linkage disequilibrium analysis using a 30 trio reference panel of Centre d'Étude du Polymorphisme Humain (CEPH) Utah Residents with Northern and Western European Ancestry (CEU) (Coriell Institute for Medical Research, Camden, NJ) was previously reported (22). LD plots were generated using Haploview (27) as previously described (22). Lymphoblastoid cell lines were grown in RPMI with 15% fetal bovine serum at 37°C in 5% carbon dioxide. RNA was isolated with Trizol (Invitrogen) and cDNA was prepared as above. To evaluate the endogenous *CD58* locus, RT-PCR was performed with primers (5-TGGTTCTGTCTGGTTTTCTGTC-3′, 5′-TGGTGTTGTGTATGGGAATGT-3′) binding in constitutive exons flanking the alternative exon and intronic polymorphism. Amplicons were run on an agarose gel and quantified as above. To quantify these results using a second method, the experiment was repeated and samples were also run on the Fragment Analyzer Automated CE System (Advanced Analytical) and quantified with the accompanying PROsize software. Similar results were obtained for quantification with peak height or peak area; data shown uses peak height. For either method, samples were stratified based on genotype and unpaired *t*-test was performed with the p-value of 0.05. We also quantified the splice variants at the endogenous locus using qRT-PCR. Primers were designed to differentiate the alternative isoforms (Supplementary Figure S2). To measure the skip of exon 3, we used a primer spanning exons 2 and 4, resulting in a product only when exon 3 was skipped. To measure the exon 3 inclusion, we used a primer that binds within exon 3. In both cases, the isoform-specific primer was paried with a shared primer in exon 2. Overall gene expression level was detected with downstream primers that would amplify the mRNA regardless of exon 3 inclusion/skip. *GAPDH* expression was another tested control; using either overall *CD58* or *GAPDH* yielded comparable results. RNA was isolated and cDNA prepared as above for seven samples with varying *Alu* genotypes (Supplementary Figure S2). qRT-PCR was performed in 20 μl reactions with 1 μl of cDNA with SsoAdvanced universal SYBR green supermix (BioRad). Reactions were initially denatured at 98°C for 30 s, and then 45 cycles of 98°C for 10 s and 60°C for 30s. Thermal dissociation curves resulted in a single clean peak for each primer pair and reaction. Ct values were determined using the standard parameters of the program. All –RT reactions had a Ct value that was not determined as no amplification was detected. For each primer pair, each sample was tested in triplicate. Relative expression of each the skip isoform and the isoform including exon 3 was calculated as 2^−ΔCt^ where ΔCt = Ct_CD58 primer pair_ – Ct_GAPDH_ within each sample. We combined results for Individuals with the same genotype and graphed them together. Depicted results are from one experiment (Supplementary Figure S2). A second experiment gave similar results.

## RESULTS

### Candidate polymorphic *Alu* elements for splicing effects

**Figure 1.**
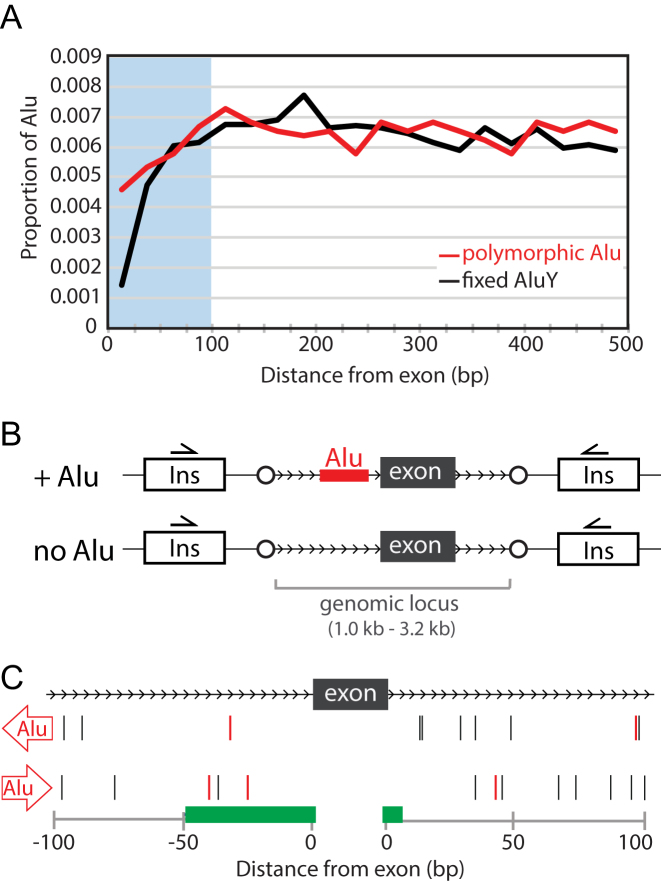
Evaluation of polymorphic *Alu* elements mapping near exons. (**A**) *Alu* elements are depleted near exons. Proportions of intronic polymorphic *Alu* elements (red) or reference *Alu*Y (black) are shown in 25 bp bins from the nearest exon. (**B**) Two constructs for each locus were evaluated using ectopic mini-gene splicing assays. The genomic locus encompassing the alternatively used exon (black) and flanking sequence was cloned into the vector containing rat insulin (Ins) exons using attP sequences (circles) and Gateway cloning (Invitrogen). (**C**) Assayed polymorphic *Alu* elements are representative of all combinations of distance and position relative to the exon and orientation relative to gene transcription. Those with effects in the ectopic assay are in red. Green bars indicate regions associated with known splicing regulatory sequence. Upstream of the exon these regulatory sequences (from left to right) include the branch site and polypyrimidine tract (the exact distance from the exon can vary, so the most common range is depicted). At the upstream exon/intron boundary is the splice acceptor site. At the downstream exon/intron boundary is the splice donor site.

To identify *Alu* elements that alter splicing, we focused on those that occur within a short distance to the nearest exon. Most sequence determinants of splicing occur proximal to exon boundaries (e.g. (28,29)), and we expected that the *Alu* elements altering splicing would most likely fall within the previously reported ‘underrepresentation zone’ that spans 100 bp 5′ and 3′ of exons (21). To confirm the depletion of *Alu* elements near exons reported by Lev-Maor *et al.* and Zhang *et al.* (20,21), we assessed the distribution of fixed-present *Alu*Y insertion sites in 25 bp bins based on distance to exons. We recapitulate the underrepresentation of *Alu* elements near exons. This depletion is evident in the regions both immediately upstream and downstream of the exon. Moreover, Zhang *et al.* also showed a depletion of the relatively recently acquired, polymorphic *Alu* insertions near exons, suggesting that this effect results from rapid selection against these insertions (21). We evaluated this using a recently updated, more inclusive catalog of polymorphic *Alu* elements (8), and we also confirmed the depletion of polymorphic *Alu* insertion variants within this 100 bp underrepresentation zone (Figure 1A). This depletion is not absolute, however; in all, we identified 168 polymorphic *Alu* elements that map within 100 bp of an exon (Supplementary Table S3).

To identify polymorphic *Alu* sequences that affect exon usage, we focused on insertion variants near alternatively spliced exons. We considered both exons that are skipped and those with altered splice sites. We found 73 polymorphic *Alu* elements within 100 bp of an alternatively spliced exon, which is not significantly different from the expected rate (*P* = 0.3125, chi square test). However, these were of particular interest to us because we hypothesized that the polymorphic *Alu* element may be a determinant of splice-site choice. Further, as many of these exons are of lengths not divisible by three, isoforms including or excluding these exons would likely result in a frameshift (*n* = 41).

Polymorphic *Alu* elements near exons share features typical of polymorphic *Alu* elements genome-wide. Most of these *Alu* elements are full length or nearly full length (270 bp average plus the polyA tail, range 72–284 bp), with only two of these *Alu* variants having significantly shorter sequence (i.e. the *Alu* variants at *DRAM1* and *P2RX7*). Also as expected, the majority of these elements are from the youngest, most commonly polymorphic *Alu* subfamilies (57.5%), including *Alu*Ya5, *Alu*Yb8 and *Alu*Yb9. Although more of these elements are anti-sense with respect to the gene, 42 compared to 31 that are sense with respect to the gene, this is not significantly different from expected (*P* = 0.1979, chi square test). Similarly, these polymorphic *Alu* elements map equally upstream (*n* = 40) and downstream (*n* = 33) of alternatively used exons (*P* = 0.671, chi-square test).

### Polymorphic *Alu* elements can affect exon usage

To determine if presence of each *Alu* variant alters incorporation of its nearby exon, we evaluated exon splicing in an ectopic minigene-splicing assay (e.g. (26,30)). We used the pSpliceExpress vector, which enables cloning of genomic loci into the minigene reporter by recombination for higher throughput (26). This vector contains two constitutive rat insulin exons flanking an intron into which we cloned the sequences to be tested, the exon(s) and surrounding intronic sequences with and without corresponding *Alu* variant (Figure 1B). We evaluated transcript isoforms after expression of the minigene reporter in 293T cells, using RT-PCR primers specific to the constitutive rat insulin exons. In all, we tested 23 loci where a polymorphic *Alu* element mapped within 100 bp of an exon (Supplementary Table S1). In selecting loci, we captured examples of every combination of *Alu* orientation and position relative to the gene and nearby exon (Figure 1C) and a variety of common *Alu* subfamilies (Supplementary Table S3). We identified significant effects of the *Alu* insertion on exon usage at five of these loci (data from those without significant effects are in Supplementary Figure S3).

**Figure 2.**
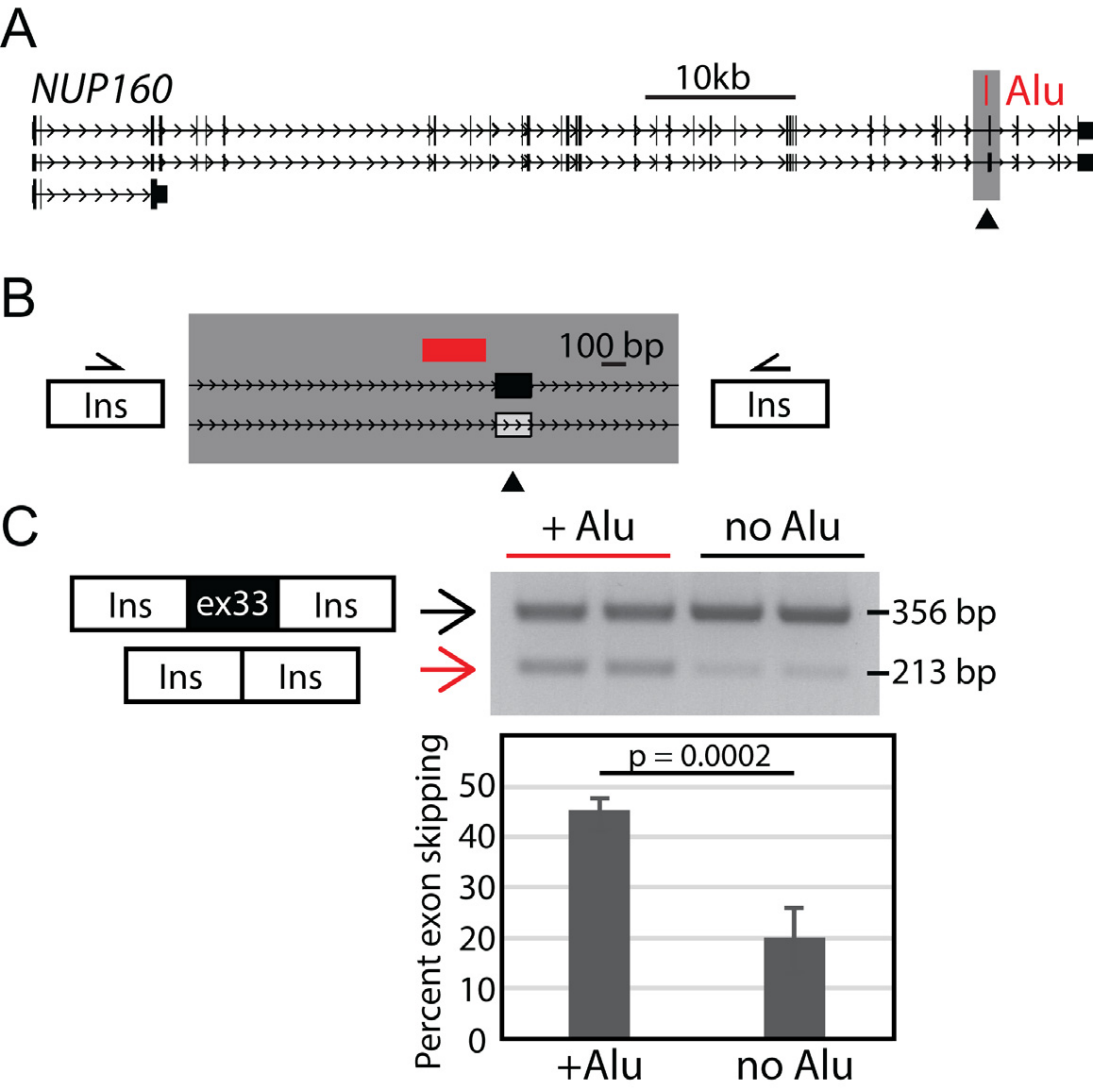
Polymorphic *Alu* results in increased exon skipping at *NUP160*. (**A**) *NUP160* locus. A polymorphic *Alu*Yh3a3 (red) is sense with respect to *NUP160* transcription and maps 41 bp upstream of alternatively used exon 33 (arrowhead). The region included in the splicing reporter is highlighted in gray. (**B**) Spicing reporter assay of *Alu* insertion effects at *NUP160* locus. Minigene-splicing reporters contain the genomic segment (gray box, also from A) with and without the *Alu* present (red). White box indicates exon is skipped in rare isoforms according to EST data. (**C**) A representative gel showing splicing assay amplicons from two independent clones for each construct assayed. Two bands of indicated sizes were quantified on an agarose gel after PCR with primers in the rat insulin (Ins) exons. The larger product incorporates *NUP160* exon 33. Data were combined with a second replicate and graphed. Error bars are the standard deviation of the four values for each construct. Unpaired *t*-test results are shown.

At one locus where we detected an effect, a polymorphic *Alu* element maps 41 bp upstream of exon 33 of the *NUP160* gene (Figure 2A). *NUP160* encodes Nucleoporin 160, a member of the 120-MD nuclear pore complex that mediates nucleoplasmic transport. Exon 33 of this gene is a near constitutive exon, but EST data (JD448821) suggest that it is skipped in a minor transcript isoform; skipping of the 143 bp exon 33 would result in a frameshift in the mRNA open reading frame. The 262 bp *Alu*Yh3a3 element at *NUP160* is oriented antisense with respect to the gene. To determine its effect on exon usage, we tested a 1743 bp fragment of this locus, both with and without the *Alu* element present (Figure 2B), in the minigene reporter assay. We detect two different splice events with both constructs. Sanger sequencing of the RT-PCR products confirmed that one event includes the *NUP160* exon 33 and the other skips the *NUP160* exon. Both spliced products are detected with and without the *Alu* insertion; however, when the *Alu* is present, the exon is skipped significantly more often, 45.2%, compared to only 20% when the *Alu* is not present (*P* < 0.001) (Figure 2C, Supplemental Figure S1). This indicates that at least in the reporter assay this *Alu* polymorphism has an effect on exon usage; the presence of the *Alu* promotes exon skipping.

### Polymorphic *Alu* elements can promote exon skipping or exon inclusion

**Figure 3.**
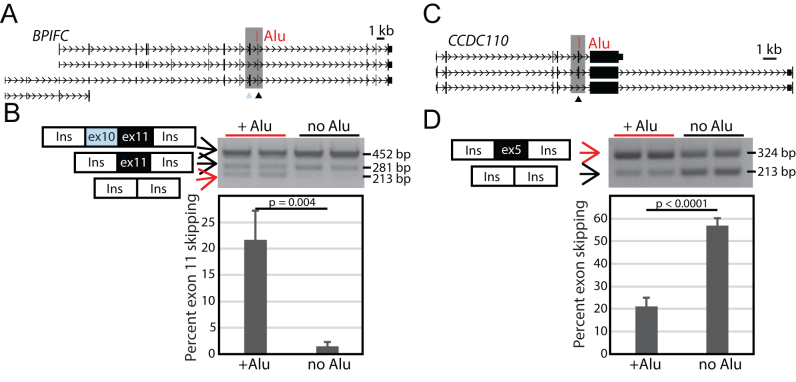
Polymorphic *Alu* elements can increase exon skipping or inclusion. (**A**) *BP1FC* locus. A polymorphic *Alu*Ya5 (red) is sense to the *BP1FC* gene and maps 25 bp upstream from alternatively used exon 11 (black arrowhead). There is also an alternatively used exon 10 (blue arrowhead) within the region that was evaluated in the splicing reporter (gray box). (**B**) A representative gel showing splicing assay amplicons from two independent clones for each construct assayed. Three spliced products were detected. Data were combined with a second replicate and the percent exon skipping of exon 11 is graphed with error bars indicating the standard deviation of the 4 values for each construct; the *Alu* results in increased exon skipping. (**C**) *CCDC110* locus. A polymorphic *Alu*Y (red) is sense to the *CCDC110* gene and maps 42 bp downstream of alternatively used exon 33 (black arrowhead). (**D**) Splicing assays were performed and are shown as in (B). At this locus, the polymorphic *Alu* element increases exon inclusion. Unpaired *t*-test results are shown.

We identified 3 other polymorphic *Alu* elements that increase skipping of nearby exons similar to that observed at *NUP160*. One example is a polymorphic *Alu* element at 22q12.3 in *BPIFC* (Figure 3A). This gene encodes a BPI Fold Containing Family C protein that binds lipids and lipopolysaccharides. There are 8 alternatively used exons within this gene. One of these, exon 11 of the transcript, which is rarely skipped, maps 25 bp from a 261 bp polymorphic *Alu*Ya5 element (Figure 3A). The polymorphic *Alu*Ya5 is oriented in sense with respect to *BPIFC*. To test the effect of this *Alu* on mRNA splicing, we cloned a 2252 bp fragment encompassing the interval of *BPIFC* exons 10 and 11, both of which are alternatively incorporated exons, and flanking intronic sequences with and without the *Alu* element (Figure 3A). We detected three bands in the ectopic assay, and Sanger sequencing confirmed these bands correspond to inclusion of both exons, only exon 11, and neither exon (Figure 3B); inclusion of only exon 10 in the absence of exon 11 was not detected, consistent with annotated isoforms. We detected a notable increase in the skipping of both exons 10 and 11 in the presence of the *Alu* element. With the *Alu*, 21.6% of transcripts skip both exons 10 and 11 compared to only 1.6% of transcripts which skip these exons when the *Alu* is absent (*P* = 0.004) (Figure 3B, Supplemental Figure S1). Therefore, in the ectopic assay, this *Alu* polymorphism affects exon usage similarly to that at the *NUP160* locus; the *Alu* promotes exon skipping. Similarly, we identified polymorphic *Alu* elements that promote skipping of a nearby exon at two other loci, *SLC2A9* and *CD58*, which we will detail in subsequent sections.

We found that polymorphic *Alu* elements can alter exon usage in the opposite manner as well. While previous examples presented here indicate that the *Alu* elements promote exon skipping, at 4q35.1, we identified a polymorphic *Alu* element that promotes the inclusion of a nearby, annotated alternatively used exon in *CCDC110* (NM_001145411.1). This alternatively used exon 5 is 111 bp in length. An *Alu* polymorphism maps 42 bp downstream of this exon (Figure 3C). The 257 bp *Alu*Y is in the same orientation as the *CCDC110* gene, which encodes Coiled-coil Domain Containing 110 protein. We tested a 1077 bp region encompassing *CCDC110* exon 5 and flanking intronic sequence in the minigene assay with and without the *Alu* present. When the *Alu* is present, the percent inclusion for exon 5 increases from 43.2% without the *Alu* to 79% when the *Alu* is present (Figure 3D, Supplemental Figure S1) (*P* < 0.0001). Therefore, in the ectopic assay, this polymorphic *Alu* element promotes the inclusion of the nearby alternatively used exon. Together with the other examples, this indicates that polymorphic *Alu* elements can alter inclusion rates for an exon in either direction: promoting exon inclusion or exon skipping.

### A polymorphic *Alu* at *SLC2A9* promotes exon skipping in a sequence independent manner

To test the hypothesis that *Alu* polymorphisms alter mRNA splicing resulting in an effect on gene function and disease risk, we evaluated two instances where an *Alu* variant near an alternatively spliced exon occurred in proximity to a genome-wide association study (GWAS) signal (22).

**Figure 4.**
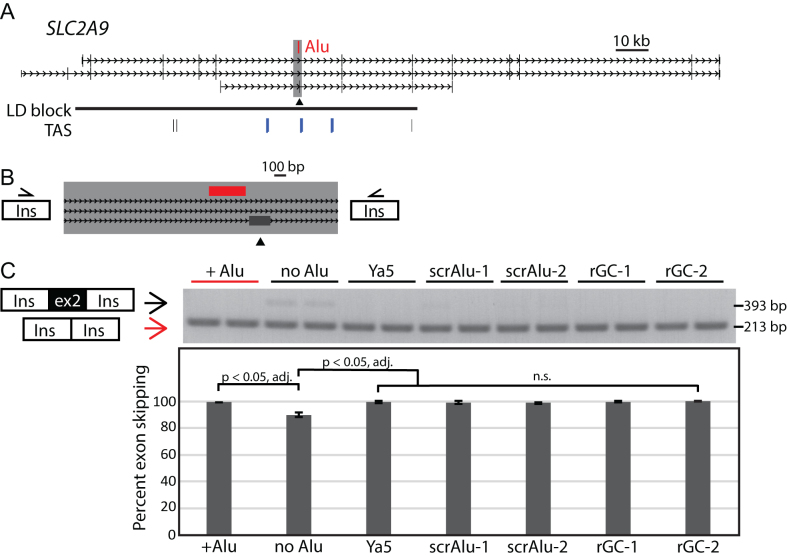
A polymorphic *Alu* increases exon skipping at *SLC2A9* in a non-sequence specific manner. (**A**) *SLC2A9* locus. A polymorphic *Alu*Yi6 (red) is antisense to *SLC2A9* transcription and maps 34 bp upstream of alternatively used exon 2 (arrowhead). An LD block (horizontal black line) spans from chr4:9529704–9634418 (hg19) is defined as a region that is in strong LD (*r*^2^ > 0.8) encompassing six trait associated SNPs (TAS, vertical lines) associated with gout and serum urate levels; three are in moderate LD with the *Alu* (blue) *r*^2^ > 0.46 *D*’ = 1 (confidence intervals 0.8–1). SNPs from left to right are rs16890979, rs4475146, rs4481233, rs7442295, rs6449213 and rs775948. (**B**) Minigene-splicing vectors contain the region encompassing exon 2 and flanking sequence with or without the *Alu* (red). (**C**) Gel of representative splicing assay results showing two independent clones for each construct assayed. Two bands of indicated size were quantified from the agarose gel. Data were combined with a second replicate and graphed with error bars indicating the standard deviation of the 4 values for each construct. Constructs with the *Alu*Yi6, *Alu*Ya5 consensus sequence, scrambled *Alu* sequence (scrAlu), and spacer sequence with GC content matching the intron (randomized sequence with controlled GC content, rGC) are not statistically different from one another (n.s.). The difference between the no *Alu* (pre-insertion) construct and all other constructs is statistically significant (unpaired *t*-tests, *P-adjusted = 0.0002).

At the first of these, a 284 bp *Alu*Yi6 maps 31 bp upstream of an alternatively used exon in the *SLC2A9* gene that encodes Solute Carrier Family 2 Member 9 protein that is involved in transmembrane transport of urate and fructose (Figure 4A). This locus has previously been associated with serum uric acid levels and risk for developing gout. Seven (7) trait-associated SNPs were reported, including rs4481233 and rs7442295 (*P* = 6 × 10^−34^ and 2 × 10^−15^) (e.g. (31,32)) which define a 105 kb linkage disequilibrium (LD) (*r*^2^ > 0.8) block. We previously reported that an *Alu* variant mapping to this interval is in moderate LD with two of these trait-associated SNPs and specifically associated with the protective haplotype (22). This moderate level of LD (*r*^2^ = 0.56, *D*’ = 1 with confidence intervals of 0.79–1) indicates that the *Alu* may cooperate with other variants on the same haplotype to affect *SLC2A9* function. To evaluate effects of this *Alu* polymorphism on splicing, we cloned a 2312 bp fragment that encompassed the alternatively used exon and *Alu* polymorphism into our ectopic splicing vector (Figure 4B). We tested the locus with and without the *Alu* present in the construct. In contrast to other loci examined, the alternative exon is part of the minor isoform and is skipped in the majority of transcripts; this is exon 2 of this isoform (DA631706) in which it is included, but it is skipped in all other isoforms. This splice pattern was recapitulated in the ectopic assay, which also showed more frequent skipping when the *Alu* is present (Figure 4C, Supplemental Figure S1). Skipping is near complete when the *Alu* is present, 99%, compared to when the *Alu* is absent, 89.9% (*P* < 0.01).

Given the clinical significance of this locus, we wanted to explore whether this suppressive effect on exon incorporation was an intrinsic feature of the *Alu* sequence (i.e., reflecting a sequence-specific effect of the *Alu*) or was caused by disruption to non-*Alu* regulatory sequences that function in the absence of the *Alu* (i.e. sequences that are intact only on the ‘empty’ allele). We hypothesized that the *Alu* could alter splicing by providing binding sites for splicing regulators (e.g. (33)) or that *Alu* RNA secondary structure (34,35) or overall GC content could regulate splice-site recognition (36). To test these models, we developed a series of reporter constructs. We replaced the *Alu*Yi6 with *Alu* sequence of a different, commonly polymorphic *Alu* subfamily (*Alu*Ya5 consensus sequence with an average size polyA tail, 5% divergent from *Alu*Yi6) or two different random scrambles of *Alu* sequence (the entire *Alu* sequence including the polyA tail was scrambled) to disrupt any protein binding sites or pre-mRNA secondary structure. We also tested the effect of two different randomized sequences of equal length to the *Alu* (including polyA tail) but with a GC content matching the integration site (46.6% GC compared to 61.6% GC for the *Alu*) (Supplementary Table S2 and methods). We found that all of these constructs resulted in >98% of transcripts skipping the *SLC2A9* exon, similar to each other and to the *Alu*Yi6 containing construct (Figure 4C, Supplemental Figure S1). Specifically, the inclusion rate of *SLC2A9* exon 2 is not statistically different for any of these constructs or the *Alu*Yi6-containing construct, but it is different relative to the construct containing no additional sequence (*P* < 0.05, adjusted for multiple testing). Thus, we conclude that the *Alu* polymorphism reduces inclusion of the alternatively used exon in *SLC2A9*, but does so independently of the specific *Alu* sequence. Since this is not a sequence-specific effect, we conclude that this *Alu* insertion variant disrupts splicing regulators that function when the *Alu* is not present.

### A polymorphic *Alu* responsible for *CD58* exon skipping may affect multiple sclerosis risk

**Figure 5.**
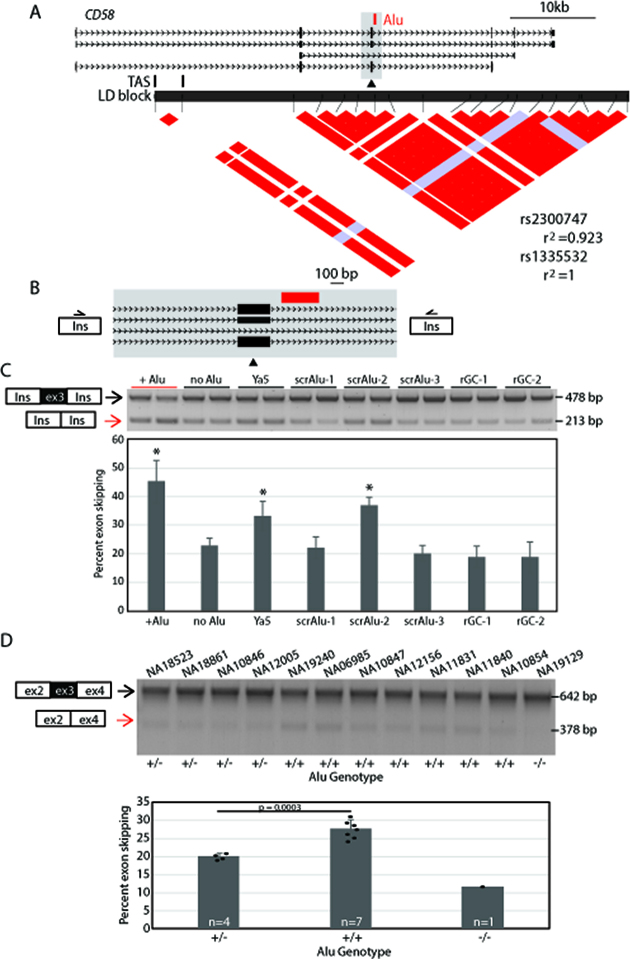
An *Alu* variant associated with MS risk is an sQTL at *CD58*. A) *CD58* locus. Trait associated SNPs (TAS, vertical black lines) identify this as a multiple sclerosis risk locus by GWAS. The LD structure from the GWAS identified LD block (black horizontal line) was generated by pair-wise comparisons of variants. Two variants in near perfect LD (*r*^2^ > 0.9) are depicted with red coloring at the intersection of the LD plot. Gray-blue boxes indicate less LD between evaluated variants. The polymorphic *Alu* element is in LD with the 2 TASs, rs2300747 and rs1335532. LD values between the TAS and the polymorphic *Alu* are shown to the right of the plot. (**B**) Minigene-splicing assay of *CD58* locus. The *CD58* genomic locus (gray box, also from A) with and without the *Alu* present (red) encompasses alternatively used exon 3. (**C**) Representative gel of splicing assay results showing 2 independent clones for each construct assayed. Two bands of indicated size were quantified from agarose gel. Data were combined with a second replicate and graphed with error bars indicating the standard deviation of the four values for each construct. The construct containing the polymorphic *Alu*Y was compared to the allele without an *Alu* present and constructs where the polymorphic *Alu*Y was replaced with the *Alu*Ya5 consensus sequence, scrambled *Alu* sequence (scrAlu), and spacer sequence with GC content matching the intron (rGC). The polymorphic *Alu*Y has the greatest effect but is not statistically different from the *Alu*Ya5 consensus or scrAlu-2. These 3 constructs (+Alu, Ya5, and scrAlu-2) are denoted with a * because they are all statistically different than the construct with the empty naturally occurring allele (no Alu). This indicates an effect of these sequences on splicing relative to the no *Alu* allele. They are also statistically different from all constructs without a * (unpaired *t*-tests, *P*< 0.05, adjusted). (**D**) Analysis of *CD58* splicing at the endogenous locus. With primers that bind in *CD58* exons 2 and 4, two spliced products, of indicated sizes, were detected at the endogenous locus. Individuals (*n* = 12) with noted *Alu* genotypes were evaluated. Graph shows quantification based on genotype with each individual indicated by a black circle. Error bars are the standard deviation within the group. An unpaired *t*-test indicates a dose dependent effect between genotypes and percent exon skipping was detected.

We previously reported an intronic *Alu* at *CD58* mapping near a GWAS signal for multiple sclerosis (MS) risk (*P* = 3 × 10^−16^) (e.g. (22,37,38)). The risk allele is associated with lower expression (39) of CD58, a cell surface protein also known as lymphocyte function-associated antigen 3 (LFA-3), that is widely expressed in hematopoietic and non-hematopoietic cells. Reduced *CD58* mRNA level is associated with both risk of developing MS and relapse of symptoms for those with MS (39). Fine mapping studies have narrowed the region to a 76 kb interval of *CD58* (39) (Figure 5A). However, the causative variant leading to this disease risk has yet to been identified. A polymorphic 281 bp *Alu*Y element maps within this region, on the risk haplotype, and is in strong LD (*r*^2^ > 0.9) with the trait-associated SNPs identified by GWAS (Figure 5A).

To test the hypothesis that the polymorphic *Alu* element is the causative variant leading to increased disease risk by altering *CD58* mRNA splicing, we investigated effects of the *Alu*Y on a nearby alternatively used exon; the AluY maps 97 bp downstream (3′) of the exon. The 265 bp exon 3 is skipped in the shortest isoform of *CD58* (uc001ego.1, FLJ77504), altering the protein reading frame. We cloned a 2340 bp fragment containing the alternatively used exon and polymorphic *Alu* insertion site into the minigene reporter and measured exon inclusion (Figure 5B). The presence of the *Alu* results in a significant decrease in incorporation of the exon, 54.7% of the transcripts include the exon when the *Alu* is present as compared to 77.2% for the pre-insertion allele (Figure 5C, Supplemental Figure S1) (*P* < 0.05, adjusted). Therefore, presence of the *Alu* results in more exon skipping.

To further dissect the mechanism by which the *Alu* alters exon splicing, we replaced the *Alu* in the *CD58*-containing minigene reporter with *Alu*Ya5 subfamily consensus sequence (*CD58 Alu* is 2% divergent from *AluYa5*, Supplementary Figure S4), scrambled *Alu* sequences (62.6% GC), and randomized sequences of the same length matching the GC content of the pre-insertion intron (35.6% GC) (Supplementary Table S2). The polymorphic *Alu*Y sequence had the greatest effect on exon skipping of all constructs tested, with 45.3% of transcripts skipping exon 3 in the presence of the *Alu* (Figure 5C). The *Alu*Ya5 consensus sequence also resulted in a significant increase in exon 3 skipping relative to the empty allele, containing no *Alu* sequence (*P*<0.05, adjusted). The polymorphic variant at *CD58* differs from the *Alu*Ya5 consensus sequence by 7 nucleotides distributed over the length of the elements (Supplementary Figure S4). The presence of the polymorphism results in relatively more exon skipping as compared to the *Alu*Ya5 consensus sequence (45.3% versus 33.2%), although this difference is not significant when accounting for the multiple comparisons made (Figure 5C, Supplemental Figure S1).

We next performed minigene reporter assays replacing the polymorphic *Alu*Y sequence with randomized sequences with varying nucleotide compositions. First, we scrambled the *Alu* sequence to retain the length and GC content of the naturally occurring polymorphism, but disrupt specific sequence motifs and RNA secondary structure. We initially evaluated two scrambled *Alu* sequences but these gave significantly different results (*P* < 0.05, adjusted) with 22% (scrAlu-1) or 37% (scrAlu-2) of transcripts skipping *CD58* exon 3 when the scrambled sequences were present (Figure 5C, Supplemental Figure S1). Given the discrepant results, we tested a third version of scrambled *Alu* (scrAlu-3) and it behaved more similarly to scrAlu-1 with 20.1% of transcripts lacking exon 3. Next, we used randomized sequence that matched the GC content of the ‘empty’ allele intron (i.e. the integration site without the *Alu* insertion). Both of these random sequences (rGC-1, rGC-2) behaved similar to the construct with no *Alu* present resulting in 19% of transcripts lacking *CD58* exon 3.

Altogether, these results indicate that the polymorphic *Alu*Y insertion variant at *CD58* increases exon 3 skipping, and suggest that this effect is sequence dependent. While the naturally occurring *Alu*Y variant had the greatest effect of sequences we tested, the *Alu*Ya5 consensus sequence, and one of three scrambled *Alu* sequences also increase *CD58* exon 3 skipping.

### A polymorphic *Alu* element is a splicing QTL at *CD58*

We next wanted to evaluate haplotype effects on exon 3 splicing at the endogenous *CD58* locus. *CD58* is widely expressed in hematolymphoid cells, which is relevant to the MS disease phenotype and makes it ideal for splicing quantitative trait loci (sQTL) analysis as a large number of well-characterized, EBV-transformed lymphoblastoid cell lines (LCLs) expressing *CD58* are readily accessible. Given this, we evaluated splicing patterns of endogenous *CD58* in individuals from the Centre d’Etude du Polymorphism Humain (CEPH)/Utah Collection [CEU] HAPMAP population with differing *CD58* haplotypes. We selected LCLs from 12 individuals; consistent with the reported minor allele frequency of 0.285 (8), seven of the samples were homozygous for the *Alu* insertion, four were heterozygous for the *Alu*, and one was homozygous for the empty (pre-insertion) allele (22). We isolated RNA from the LCLs, performed RT-PCR using primers in exons flanking the alternatively used exon, and used two different methods (agarose gel and fragment analyzer) to detect and quantify *CD58* splice variants (Figure 5D, Supplementary Figure S5, methods). We identified a genotype-dependent effect on exon skipping. The presence of the *Alu* results in relatively more of the isoform lacking exon 3 (*P* = 7.96e–10, ANOVA). Further, homozygotes with two copies of the *Alu*-containing allele show greater exon skipping than heterozygotes having only one copy of the *Alu*-containing allele (*P* = 0.0003, *t*-test). Within one genotype, no significant differences were seen in percent exon skipping (*P* > 0.05, not significant, adjusted). To confirm this result, we used qRT-PCR to measure transcript isoforms independent of each other in a subset of the LCLs. We designed one primer pair to amplify only the exon 3-containing transcript (one primer binds within exon 3) and a second primer pair to selectively amplify the isoform without exon 3 (one primer spans the exon 2- exon 4 junction) (Supplemental Figure S2). We measured the ratio of the two isoforms and found there is more exon skipping in the presence of the *Alu* (*P* < 0.05, unpaired *t*-test) (Supplemental Figure S2). All of these results are in agreement with the ectopic minigene-splicing assay. Altogether, our findings demonstrate genetic evidence that the *Alu* insertion promotes skipping of *CD58* exon 3.

## DISCUSSION

Splicing and processing mRNA transcripts are key aspects of gene function, and genetic variants affecting these can be critical determinants of phenotypes (40,41). Sequences that regulate splicing are complex and not fully understood (e.g. (29)) and *Alu* insertions can alter their activities (11,13,19,20). In this report, we sought to delineate the impact of polymorphic *Alu* elements on alternative splicing. To this end, we evaluated 23 polymorphic *Alu* elements near exons for effects on exon splicing. All mapped within 100 bp of an exon, a zone where *Alu* elements are underrepresented genome-wide. Using a minigene reporter assay, we identified five of these loci where presence of the *Alu* variant altered splicing patterns, ∼22% of loci tested. These data indicate that common *Alu* variants can have effects on exon usage. While previous reports have identified effects of *Alu* sequences on splicing in minigene-splicing assays (19,20), our report is the first systematic study of common insertion polymorphisms for this effect. We now identify commonly occurring polymorphic *Alu* elements associated with altered exon incorporation rates.

That polymorphic *Alu* elements alter splice site choice may have physiological implications including as part of the common variant, common disease paradigm. Previously, we reported that a subset of polymorphic *Alu* elements may be functional variants contributing to disease risk detected by GWAS (22). Now, we demonstrate a mechanism by which this may occur. The polymorphic *Alu* element at the *CD58* locus is in near-perfect LD with trait-associated SNPs identified for MS risk. Here, we demonstrate that the risk allele is a sQTL at *CD58*, and that the *Alu* promotes *CD58* exon 3 skipping. This is predicted to result in a frame shifted protein, likely related to the reduced *CD58* expression reported in MS patients (39).

Our results indicate that the mechanisms by which *Alu* elements alter mRNA isoform abundance are complex and locus-specific. We identified polymorphic *Alu* elements affecting splicing for elements both upstream and downstream of the alternative exon, sense and antisense to the gene, and at varying distances from the intron–exon junction (Figure 1C). Given that no *Alu* characteristic(s) was common to only *Alu* elements that effect splicing, we considered some genic features that may sensitize a locus to *Alu*-dependent alterations in splicing. We considered the lengths of both the alternatively spliced exon and the adjacent, *Alu*-containing intron. In both cases, these intervals did not differ between loci with and without an *Alu* effect (data not shown). We also considered that the *Alu* might only have an effect on exons with weak splice sites. However, we detect effects at loci with strong (e.g. *CD58*) or weak splice sites (e.g. *SLC2A9*). It is likely that some combination of *Alu* and genic sequences determine the impact (if any) of an *Alu* on splicing rates for a nearby exon. Because of the limited number of loci evaluated, we are not able to decipher the key regulatory code. Further, even among the loci where an *Alu* effected splicing, the direction and degree of that effect also varied; while the typical exon-proximal *Alu* element promotes exon skipping (Figures 2, 4 and 5), at one locus, the presence of the *Alu* element *increases* exon inclusion (Figure 3C and D).

Whether a polymorphic *Alu* element alters exon incorporation by disrupting or delivering key regulatory sequences also appears locus-dependent. We built a series of reporter constructs at two loci to address this question. At one, *SLC2A9*, randomized sequences had a similar effect on exon skipping indicating the *Alu* insertion likely has a disruptive effect (Figure 4). Consistent with this, the polymorphic *Alu* is located within a region that is replete in splicing regulatory sequences (i.e., within 40 bp upstream of an exon) (19). We suspect that many polymorphic *Alu* elements that fall within this region would have an effect on splicing by disrupting the relative positioning of key splicing sequences. Notably, however, an *Alu* insertion location relative to an exon is not a consistent predictor of the effect of the *Alu* on splicing patterns. While 3 of the *Alu* elements that induce exon skipping are a similar distance upstream of the exon, another locus with a polymorphic *Alu* element mapping to the same region did not have altered exon usage (Figure 1C).

In other cases, the *Alu* sequence itself may play a more sequence-specific role, such as at *CD58* where the *Alu*Y insertion has an effect on exon usage not recapitulated by all randomized sequences tested (Figure 5C). As the *CD58* polymorphic *Alu*Y maps almost 100 bp downstream of the alternatively used exon, it emphasize the longer-range effects of these structural variants on exon incorporation. Here, it appears that this *Alu* introduces sequences that promote exon skipping (Figure 5C). Native *Alu* sequences at this site may recruit splicing suppressor(s) or create secondary RNA structures that sterically hinder the splicing reaction. It is possible that at *CD58*, these mechanisms combine with others since scrambled *Alu* sequences inconsistently and partially recapitulate the effect (scrAlu-2, Figure 5C). These scrambled sequences likely remove specific protein binding sites and relieve significant secondary structures associated with *Alu* elements. Here, this *Alu* introduces sequences that suppress splicing and increases exon skipping (Figure 5C). Because *Alu* sequences contain cryptic splice sites, they can become exonized and part of the mature transcript (e.g. (4,15,16)). To suppress this exonization and maintain transcript integrity, hnRNP C binds to *Alu* elements preventing their exonization by competing with the splicing factor U2AF65 (33). While we do not detect exonization of the *Alu* at any of the loci evaluated here, this mechanism may explain the increased rate of exon skipping of exons nearby an *Alu* element. At loci with an *Alu* sequence dependent effect, such as *CD58*, the suppression of the *Alu* exonization may ‘spread’ beyond the limits of the *Alu* sequence, limiting the access of splicing factors to the nearby exon, resulting in additional exon skipping.

The minigene-splicing assay appears to reproduce genotype-dependent splicing patterns at the endogenous *CD58* locus. The relatively high expression level of this gene in lymphoblastoid cell lines allowed us to evaluate splicing isoforms of the endogenous *CD58* gene in individuals with various *Alu* variant genotypes. Our results demonstrated an *Alu*-allele dose-dependent increase in exon skipping. We were particularly interested in this locus because the *Alu*-containing *CD58* haplotype is associated with MS risk, and decreased expression of CD58 has been associated with both disease risk and recurrence (e.g. (31,32)). Our results show that presence of the *Alu* on the risk allele results in increased exon 3 skipping, and that this effect is specific to this *Alu* sequence. Interestingly, increased skipping of an alternative exon of another gene, *IL7R*, has also been implicated in MS risk (e.g. (42,43)). The mechanism is different in these two cases. The *IL7R* alternative splice event reflects an interaction between *cis*-acting sequence variants and the *trans*-acting DDX39B regulator (43); DDX39B does not regulate *CD58* splicing. Further studies will be necessary to fully elucidate how altered *IL7R* and *CD58* gene functions contribute to MS risk.

Altogether, this work reveals a prevalent regulatory effect of polymorphic *Alu* elements mapping near alternative exons. At 22% of evaluated loci, we identified altered exon incorporation rates dependent on the *Alu* genotype. Further, it is likely that this is only part of the story since our experimental approach is not designed to capture tissue-specific or long-distance effects. It is well documented that mRNA isoform abundance can vary significantly between tissue types and developmental time points (e.g. (44–47)). While studies relating genotype and phenotype often encompass exonic variants, our work highlights the importance of also considering transcript structure and characterizing sequence variants adjacent to exons.

## Supplementary Material

Supplementary Data
