# Supplementary material for: Alu insertion variants alter mRNA splicing
Source: Nucleic Acids Res. 2018 Nov 10;47(1):421–31. doi: 10.1093/nar/gky1086 (PMC6326789; doi:10.1093/nar/gky1086)
Supplement: Supplementary Data [file gky1086_supplemental_files.zip › Payer_Supp_combined.pdf]

SUPPLEMENTARY MATERIAL

Figure Legends:

Figure S1. Minigene assays identify effects at 5 loci. Quantification of each replicate for all constructs evaluated for the 5 loci where significant effects were attributable to the polymorphic *Alu* element are shown. The tested constructs are labeled as in Figures 2-4: scrAlu constructs contain scrambled *Alu* sequence and rGC constructs contain randomized sequence that matches the GC content of the intron. For each construct 2 independent clones (labeled A and B) were evaluated in two separate experiments. Results shown in Figures 2-5 are the combination of these 4 data points.

Figure S2. qRT-PCR confirms sQTL at *CD58*. A) Assay design. Cartoon of CD58 transcript with exons (boxes) labeled with exon number. The two major variants (with and without exon 3) are shown. qRT-PCR primers (numbered, shown above exons) were designed to measure either the variant containing exon 3 (blue primers) or the transcripts skipping exon 3 (orange primers). An additional primer set (black) amplifies the gene regardless of variant. Primer sequences are provided for the numbered primers. B) qRT-PCR results are shown as the relative expression ($2^{-\Delta Ct}$ where $\Delta Ct = Ct_{CD58\ primer\ pair} - Ct_{GAPDH}$) of the skip event (blue primers in A) or for the inclusion event (orange primers in A). Quantification of 7 samples (black circles) with differing *Alu* genotypes is shown. Quantification was pooled together based on genotype: homozygous present for the *Alu* (+/+, NA10854, NA11840, NA19240), heterozygous for the *Alu* variant (+/-, NA12005, NA18523, NA18861), or homozygous for the pre-insertion empty allele (-/-, NA19129). Unpaired t-test p value is given.

Figure S3. *Alu* elements did not affect the rate of nearby exon skipping at 18 loci. Using the minigene assay, 18 loci with polymorphic *Alu* elements were eliminated from further analysis because the percent exon skipping was statistically the same for the *Alu* containing construct (+) and the construct without the *Alu* present (-). Error bars are the standard deviation of 4 values for each construct. Unpaired t-test results are shown.

Figure S4. The alignment between the *CD58 Alu*Y sequence and the *Alu*Ya5 consensus sequence. Sequence comparisons show the sequences differ by 7 bp. Of these, 5 (pink) are those that define the *Alu*Ya5 subfamily and 2 (blue) are positions where the sequence of the CD58 *Alu*Y element deviates from the consensus sequences in a non-subfamily specific way.

Figure S5. Secondary method to quantify effects confirms *Alu* variant is a sQTL at *CD58*. The isoforms detected from endogenous CD58 were evaluated in a subset of lymphoblastoid cell lines (n=9) using fragment analyzer (see methods) A) Examples of fragment analyzer traces. The upper-most and lower-most peaks are the size markers (M) used in analysis. The two peaks are the two PCR products with and without incorporation of exon 3. A zoom in on these two peaks is shown for one sample with the peak height above baseline indicated. C) Quantification of 9 samples (black circles) with differing *Alu* genotypes is shown. Quantification was pooled together based on genotype: homozygous present for the *Alu* (+/+, NA06985, NA10854, NA11840, NA19240), heterozygous for the *Alu* variant (+/-, NA10846, NA12005, NA18523, NA18861), or homozygous for the pre-insertion empty allele (-/-, NA19129). Unpaired t-test p value is given.

TABLE LEGENDS:

Table S1. 23 polymorphic *Alu* elements evaluated in the minigene reporter assay with primer sequences. Locations of polymorphic Alu elements annotated as reported in (1). TSD=target site duplication. *Alu* allele frequency as reported in (1) and (2) when available within different reference populations (all 1000 genome populations, EUR super population, or CEU subset).

Table S2. Additional sequences tested in ectopic assays of *SLC2A9* and *CD58* loci.

Table S3. 168 polymorphic *Alu* elements within 100 bp of intron-exon boundary.
Locations of polymorphic Alu elements annotated as reported in (1).

REFERENCES:

1. Sudmant, P.H., Rausch, T., Gardner, E.J., Handsaker, R.E., Abyzov, A., Huddleston, J., Zhang, Y., Ye, K., Jun, G., Fritz, M.H. *et al.* (2015) An integrated map of structural variation in 2,504 human genomes. *Nature*, **526**, 75-81.
2. Payer, L.M., Steranka, J.P., Yang, W.R., Kryatova, M., Medabalimi, S., Ardeljan, D., Liu, C., Boeke, J.D., Avramopoulos, D. and Burns, K.H. (2017) Structural variants caused by Alu insertions are associated with risks for many human diseases. *Proc Natl Acad Sci U S A*, **114**, E3984-E3992.

FigureS1

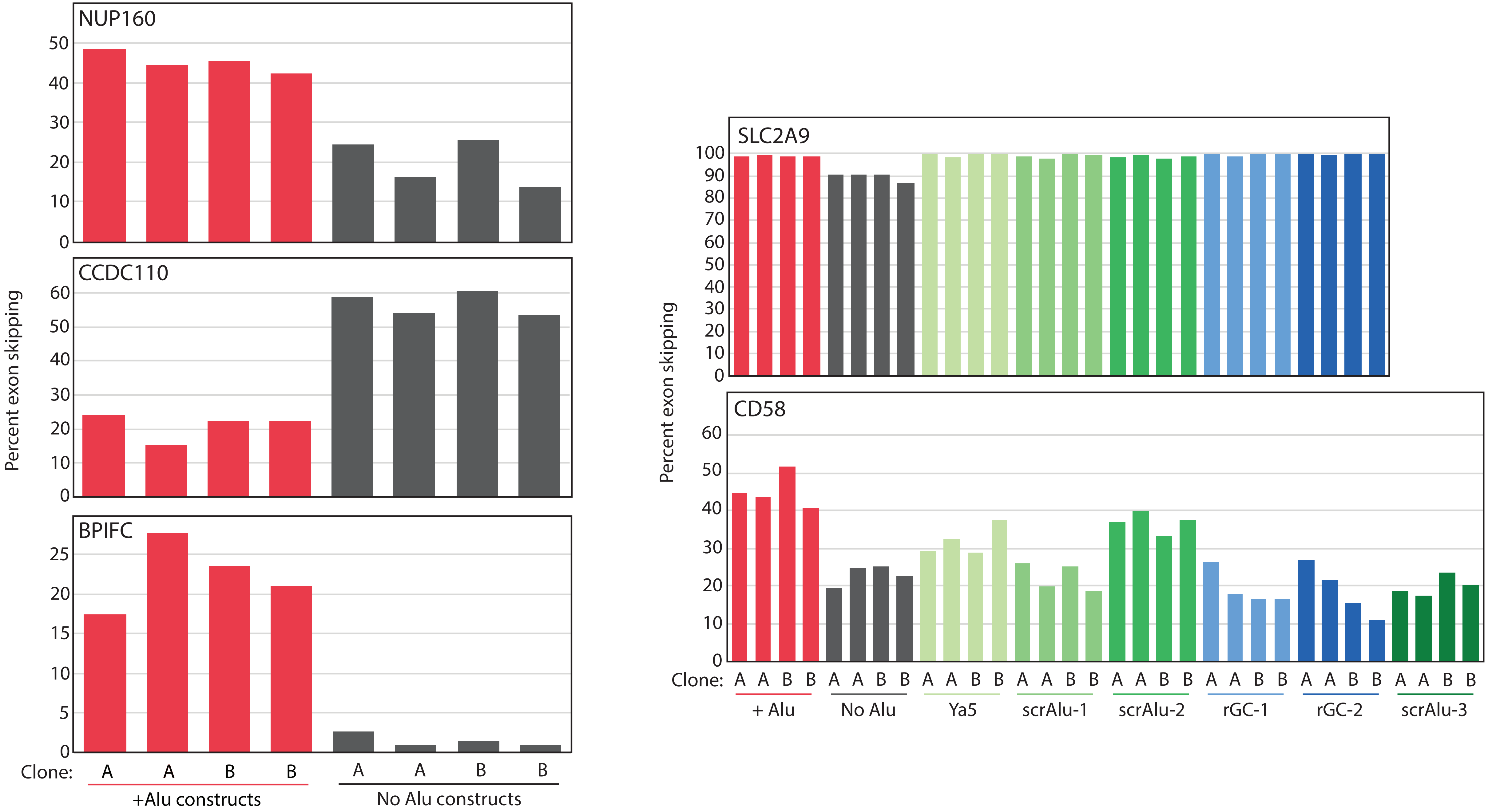

NUP160
CCDC110
BPIFC
SLC2A9
CD58
Percent exon skipping
Clone:
A A B B A A B B
+Alu constructs
No Alu constructs
+ Alu
No Alu
Ya5
scrAlu-1
scrAlu-2
rGC-1
rGC-2
scrAlu-3

FigureS2

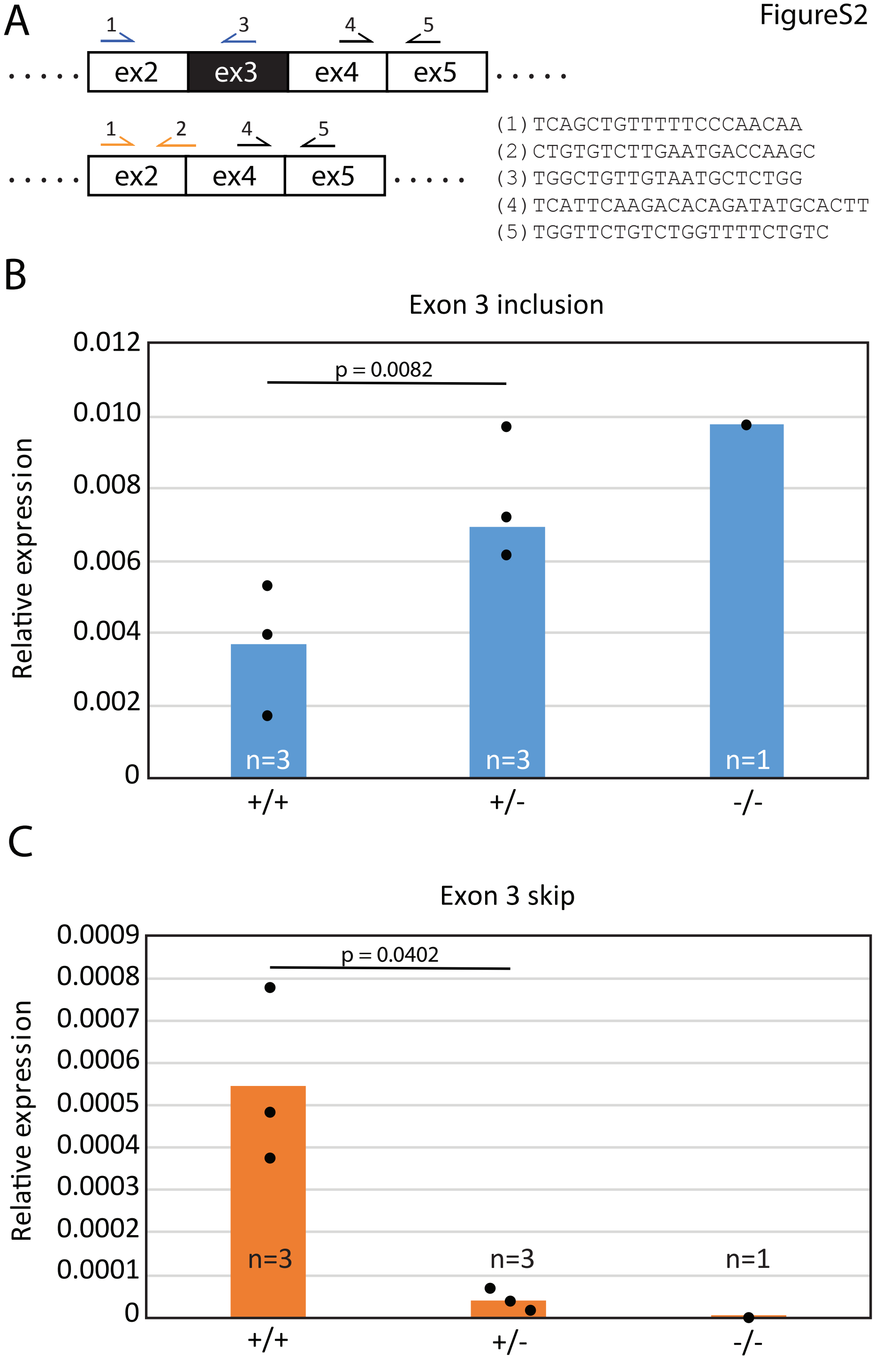
A
1
3
4
5
ex2
ex3
ex4
ex5
1
2
4
5
ex2
ex4
ex5
(1) TCAGCTGTTTTTCCCAACAA
(2) CTGTGTCTTGAATGACCAAGC
(3) TGGCTGTTGTAATGCTCTGG
(4) TCATTCAAGACACAGATATGCACTT
(5) TGGTTCTGTCTGGTTTTCTGTC
B
Exon 3 inclusion
Relative expression
0.012
0.010
0.008
0.006
0.004
0.002
0
p = 0.0082
n=3
n=3
n=1
+/+
+/-
-/-
C
Exon 3 skip
Relative expression
0.0009
0.0008
0.0007
0.0006
0.0005
0.0004
0.0003
0.0002
0.0001
0
p = 0.0402
n=3
n=3
n=1
+/+
+/-
-/-

FigureS3

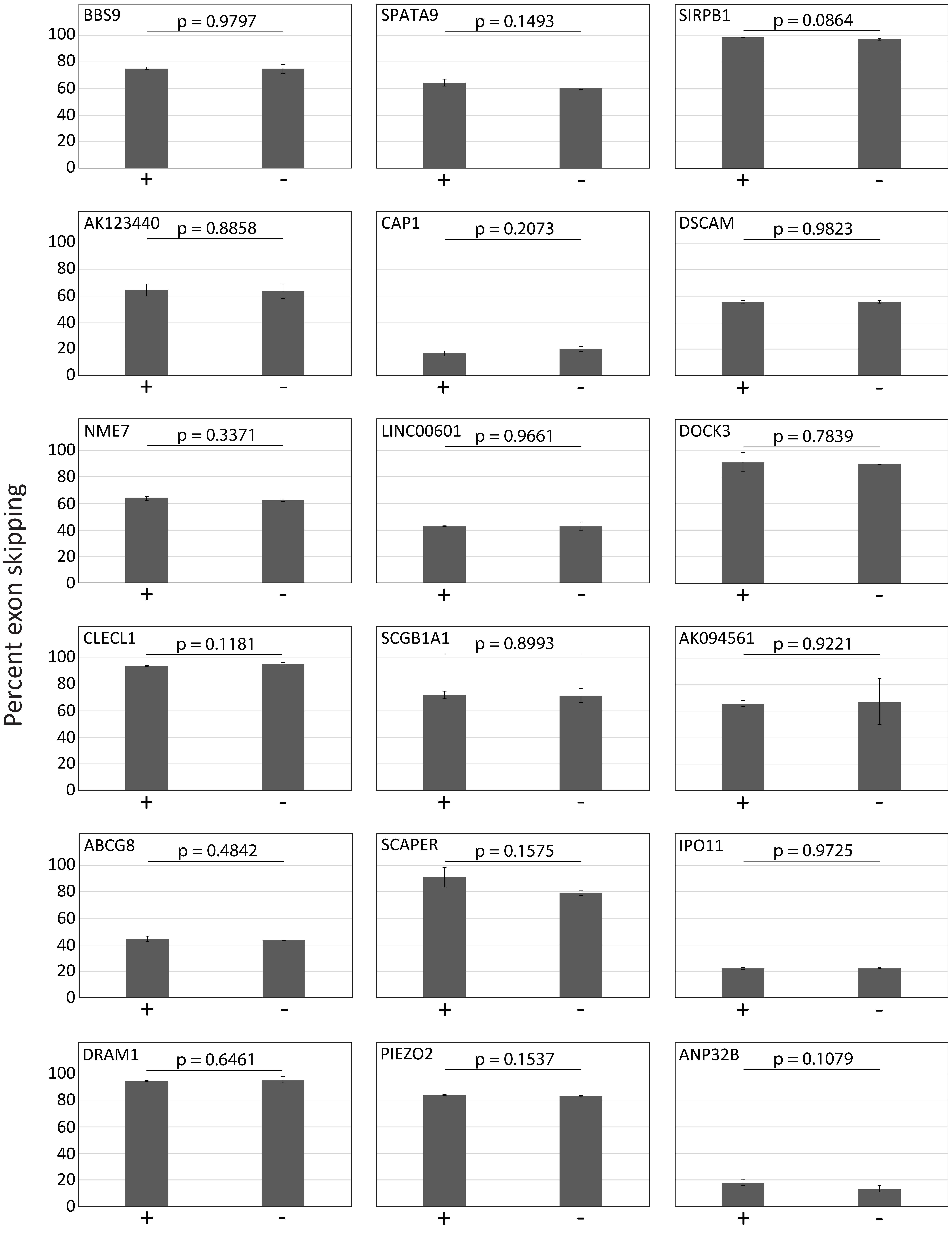
Percent exon skipping
0
20
40
60
80
100
+
-
BBS9
p = 0.9797
SPATA9
p = 0.1493
SIRPB1
p = 0.0864
AK123440
p = 0.8858
CAP1
p = 0.2073
DSCAM
p = 0.9823
NME7
p = 0.3371
LINC00601
p = 0.9661
DOCK3
p = 0.7839
CLECL1
p = 0.1181
SCGB1A1
p = 0.8993
AK094561
p = 0.9221
ABCG8
p = 0.4842
SCAPER
p = 0.1575
IPO11
p = 0.9725
DRAM1
p = 0.6461
PIEZO2
p = 0.1537
ANP32B
p = 0.1079

FigureS4

Score	Expect	Identities	Gaps	Strand
518 bits(280)	9e-152	294/301(98%)	0/301(0%)	Plus/Plus

```
CD58 1     GGCCGGGCGCGGTGGCTCACGCCTGTAATCCCAGCACTTTGGGAGGCCGAGGCGGGCGGA  60
           ||||||||||||||||||||||||||||||||||||||||||||||||||||||||||||
Ya5  1     GGCCGGGCGCGGTGGCTCACGCCTGTAATCCCAGCACTTTGGGAGGCCGAGGCGGGCGGA  60

CD58 61    TCACGAGGTCAGGAGATCGAGACCATCCTGGCTAACACGGTGAAACCCCGTCTCTACTAA  120
           |||||||||||||||||||||||||||| |||||| ||||||||||||||||||||||||
Ya5  61    TCACGAGGTCAGGAGATCGAGACCATCCCGGCTAAAACGGTGAAACCCCGTCTCTACTAA  120

CD58 121   AAATACAAAAAATTAGCCGGGCGTGGTGGCGGGCGCCTGTAGTCCCAGCTACTCGGGAGG  180
           |||||||||||||||||||||||| |||||||||||||||||||||||||||| ||||||
Ya5  121   AAATACAAAAAATTAGCCGGGCGTAGTGGCGGGCGCCTGTAGTCCCAGCTACTTGGGAGG  180

CD58 181   CTGAGGCAGGAGAATGGCGTGAACCCAGGAGGCGGAGCTTGCAGTGAGCCGAGATCGCGC  240
           |||||||||||||||||||||||||| ||||||||||||||||||||||||||||| |||
Ya5  181   CTGAGGCAGGAGAATGGCGTGAACCCGGGAGGCGGAGCTTGCAGTGAGCCGAGATCCCGC  240

CD58 241   CACTGCACTCCAGCCTGGGCGACAGAGCGAGACTCTGTCTCAAAAAAAAAAAAAAAAAAA  300
           ||||||||||||||||||||||||||||||||||| ||||||||||||||||||||||||
Ya5  241   CACTGCACTCCAGCCTGGGCGACAGAGCGAGACTCCGTCTCAAAAAAAAAAAAAAAAAAA  300

CD58 301   A  301
           |
Ya5  301   A  301
```

A

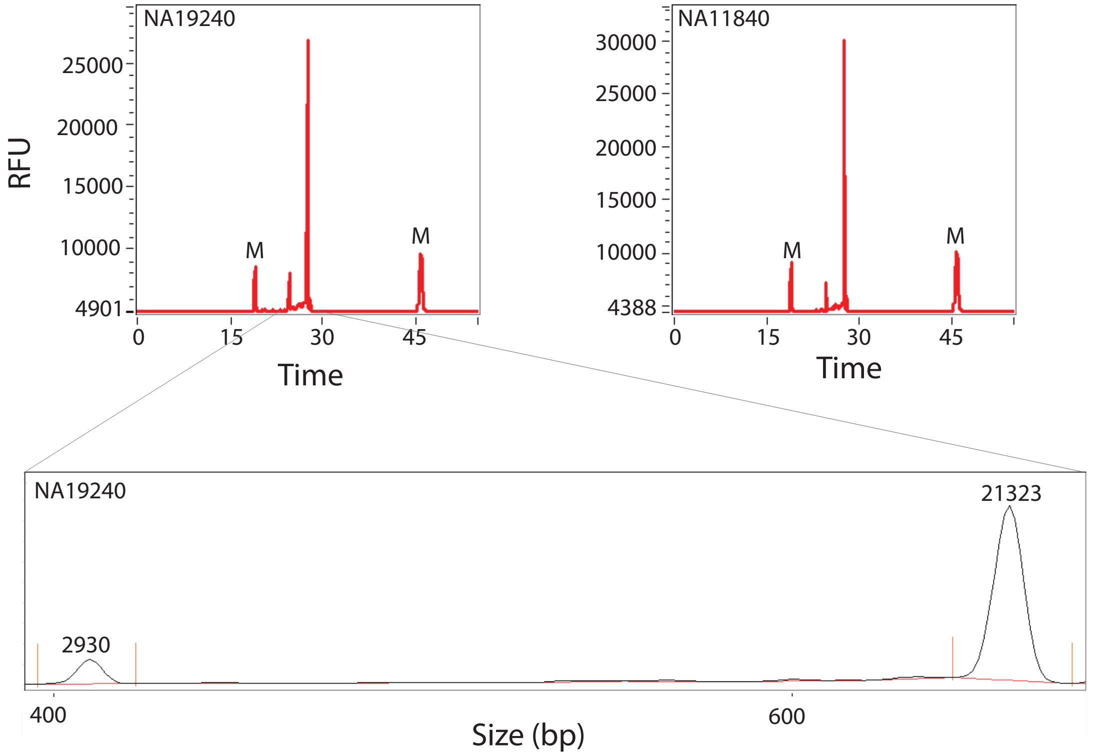
NA19240
RFU
25000
20000
15000
10000
4901
M
M
0
15
30
45
Time
NA11840
30000
25000
20000
15000
10000
4388
M
M
0
15
30
45
Time
NA19240
2930
21323
400
600
Size (bp)

B

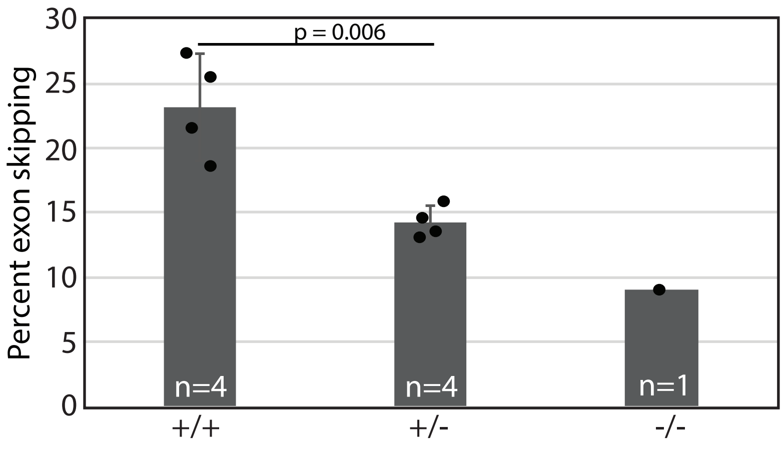
Percent exon skipping
30
25
20
15
10
5
0
p = 0.006
n=4
n=4
n=1
+/+
+/-
-/-
